# Occurrence of Microplastics in Most Consumed Fruits and Vegetables from Turkey and Public Risk Assessment for Consumers

**DOI:** 10.3390/life13081686

**Published:** 2023-08-04

**Authors:** Rana Berfin Aydın, Aykut Yozukmaz, İdris Şener, Funda Temiz, Daniela Giannetto

**Affiliations:** 1 Department of Biology, Faculty of Science, Muğla Sıtkı Koçman University, Muğla 48000, Turkey; ranaberfin256@gmail.com (R.B.A.); tmzfunda@gmail.com (F.T.); 2 Department of Aquatic Sciences, Faculty of Fisheries, Muğla Sıtkı Koçman University, Muğla 48000, Turkey; aykutyozukmaz@mu.edu.tr (A.Y.); idris_943@hotmail.com (İ.Ş.)

**Keywords:** human health risk, microplastic, Estimated Annual Intake, Estimated Daily Intake

## Abstract

Microplastics are transferred to humans through the food chain by consuming food contaminated with microplastics. However, the knowledge about the risks of dietary exposure for humans to these particles is very limited. Moreover, only a few studies on microplastic pollution in fruit and vegetables have been carried on. Thus, this study aims to investigate the presence of microplastics in some of the most consumed fruits and vegetables (pear (*Pyrus communis*), apple (*Malus domestica*), tomato (*Solanum lycopersicum*), onion (*Allium cepa*), potatoes (*Solanum tuberosum*), and cucumber (*Cucumis sativus*)) from Turkey and to evaluate the potential risk for consumers. Fruits and vegetable samples were purchased from different markets and fruiterer (two of each) in Muğla province, Southwest of Turkey. Microplastic extraction processes were carried out on the edible parts of the samples. According to the results obtained, a total of 210 particles (2.9 ± 1.6 particle g^−1^) were detected in all samples. Any significant difference occurred among the different markets. The maximum average amount of microplastic was determined in tomato samples (3.63 ± 1.39 particle g^−1^). The highest microplastic intake was with tomato (398,520 particles individual^−1^ year^−1^ for Estimated Annual Intake (EAI) and Estimated Daily Intake (EDI) for children 68.24 particles kg^−1^ day^−1^). The occurrence of microplastics of big size, that are not allowed to pass by plant xylem transport, suggests that fresh vegetables and fruits can be contaminated with plastic, especially during the production phase, during agricultural activities and during the marketing process (transport to the market and purchasing process).

## 1. Introduction

Plastic is a range of polymer materials that, due to its light weight, durability, low cost, and low thermal conductivity, have been rapidly increasing in production and use in the last 50 years. Global plastic production is shaping increasing passing from an approximately 1.5 million tons in the 1950s [1] to 390.7 million tons in 2021 [2]. Although the ease of production and use of plastics is regarded as an advantage for humanity, the most important disadvantage in terms of environment is that they gain waste status after the end of their usage span [3]. Increasing population and consumption habits lead to the emergence of plastic waste in amounts that cannot cope. Due to the mismanagement of plastic waste, approximately one-third of them leakage into both terrestrial and aquatic ecosystems and pollute the environment [4]. As a result, over the past decade, plastic debris in both marine and freshwater systems has become an emerging issue [5]. Studies have revealed that almost 80% of marine litter is made up of plastics [6,7].

Plastics entering the ecosystem as waste is exposed to degradation as a result of the effect of natural processes and environmental factors (mechanical degradation such as wind and erosion, UV radiation, biological degradation, etc.) and decompose into smaller particles, and they are transported into the human metabolism by being involved in the food chain [8,9,10,11,12,13]. Thus, according to their size, plastics are categorized into two different categories: plastic materials with dimensions larger than 5 mm are defined as “Macroplastic”, whereas “small pieces of plastic between 0.1 µm and 5 mm in diameter” are defined as “Microplastic” [14,15,16,17,18,19,20,21,22].

Microplastics were first noted in North America as spherules in plankton tows along the coast of New England in the 1970s [23] and in a study conducted on the coast of New Zealand, it was revealed for the first time in 1977 that plastic waste was a source of pollutants [24]. Although plastics entered our lives about a century ago, the fact that they have increased enough to form litter islands in the oceans shows how serious the threat is [25]. Plastic particles found in marine and freshwater ecosystems can spread over a wide area, including currents and hydrodynamic processes in the aquatic environment, and terrestrial ecosystems due to their hydrophobic properties [8,26,27].

Entering ecosystems in this way, microplastics continue to decompose constantly and exist in every abiotic part of the ecosystem, and inevitably negatively affect the natural life cycle of all living groups (plants and animals) [28,29,30]. Microplastics can both bioaccumulate and be transported to the upper levels of the food chain by entering the structure of plants and animals [28,29,31,32,33]. In addition, microplastics can cause the concentration of other toxic pollutants to increase in natural ecosystems and the transport of microorganisms to distant regions due to the adsorption on surface areas [34,35]. There is an increased interest to understand the impacts of microplastics on natural ecosystems, as the impacts still remain poorly understood.

Due to its durability, easy formability and low cost compared to other raw materials [36], plastics have started to be used intensively in every field of industry, including agricultural activities. The most commonly used plastics in agricultural activities are basically Polyethylene (PE), Polypropylene (PP), Poly-vinyl chloride (PVC), Polyethylene Terephthalate (PET), and rarely Polycarbonate (PC) type plastics. All these plastics and their outputs (MPs) remain on the soil surface during or after agricultural production and may cause pollution. These plastics remaining on the soil surface, as in other ecosystems, decompose into smaller pieces due to many different physical and biological factors, turn into microplastics and infuse into the soil [37,38]. Impacts of MPs in terrestrial ecosystems can be related to the ingestion of particles by soil organisms causing harm to their growth, biosorption through the roots of plants and reproduction at different trophic levels of the food chain and to the total environment [39,40,41].

Microplastics are transferred to humans through the consumption of food contaminated with these particles [20,42]. Although it is well-known that some additives (Bisphenol A (BPA), Phthalates, Polybrominated diphenyl ethers, etc.) used in the production of plastics have harmful effects on humans [4,43], there is still limited information on the effects of microplastics to human health and their toxicity. Nevertheless, their occurrence in the human body was concerning and reported even in the placenta [44] and in blood [45]. Despite this, there is no international food standard limit yet determined for plastic contamination control. Hence, it is very important to investigate the presence of microplastics starting from the bottom of the food chain and to evaluate the possible risks on human health. To date, the occurrence of microplastics was mainly reported on seafood (e.g., mussels, fish, and zooplankton) [46,47,48,49]. However, many other land-based foods as well as processed food were found to be contaminated with microplastics [50,51,52,53,54,55]. One of the main sources of microplastics in food originates from plastic packaging materials that come into direct contact with food items during the production processing and marketing chain [56].

With regards to fruits and vegetables, to date, only a few studies aiming to investigate the occurrence of microplastics in these highly consumed healthy food items were carried out globally [57,58,59]. With regard to Turkey, this is the first study which reports the occurrence of microplastic in agricultural foods.

Thus, this study aimed to determine the occurrence of microplastic in some of the most consumed fruits and vegetables (tomato, cucumber, onion, potatoes, apple, and pear) in Turkey and purchased from different markets and fruiterer in Muğla (Turkey). A further aim was to assess the risk in terms of public health.

## 2. Materials and Methods

### 2.1. Study Area and Sampling

Muğla, located in the Southern Western of Turkey, is one of the most important centers of beekeeping and olive cultivation in the country. In addition, fruit farming (especially citrus and pomegranate) is also carried out in the region. Fruits (apple and pear) and vegetables (tomato, cucumber, onion, and potatoes) samples were purchased from different markets (2 markets and 2 fruiterer) located in Muğla. The purchase sites of the samples were named M_1_ = Market 1, M_2_ = Market 2, F_1_ = Fruiterer 1 and F_2_ = Fruiterer 2. A number of 3 samples for each product were purchased from each market and fruiterer (12 samples for each fruit and vegetable for a total of 72 samples). The samples were transported to the laboratory and stored under cold conditions (+4 °C) until further analyses.

### 2.2. Prevention of Contamination during the Laboratory Process

To prevent external contamination of samples during the laboratory process the following precautions were taken. Only cotton aprons were worn during the analyses. All laboratory equipment was rinsed with pre-filtered distilled water in order to remove possible particles inside and stored in a fume hood. All the doors and windows of the laboratory were kept closed during the analyses to prevent airborne contamination [60,61]. In addition to all these precautions, in order to evaluate the potential microplastic contamination that could occur during laboratory studies and can interfere with the results, 4 filter papers were left at different points of the laboratory during the analyses to detect airborne contamination. The time taken for the analysis of each sample during the laboratory study was calculated as approximately 40 min (±5 min). Thus, the amount of microplastics on the control filters were counted under a stereo microscope and were deduced from all the results obtained. The average number of microplastics detected on the control filters placed in different points of the laboratory, that could interfere in a 40 min period, airborne interferences calculated as <1 for each type or color and was considered irrelevant according to [62,63,64].

### 2.3. Extraction of Microplastics from Fruit and Vegetable Samples

Fruit and vegetable samples were rinsed thoroughly with pre-filtered distilled water, then peeled and sliced using a sterile stainless steel knife on the same day. Three samples of 1 gr were taken from each item (triplicate). After the sliced fruit and vegetable samples were placed in glass beakers, the mouths of the beakers were covered with aluminum foil. Then, the beakers were left to dry in an oven set at 60 °C for 24–48 h. The dried samples were pulverized using a sterilized steel-made blender. Given the lack of a standard international protocol in the literature for the extraction of microplastics from fruits and vegetables, the methods reported by [65] modified by [59] was applied in this study. Accordingly, 5 g of dried powdered fruit and vegetable samples were weighed and placed into glass centrifuge tubes with 50 mL capacity. A total of 20 mL of prefiltered distilled water was added to each tube and the samples were centrifuged at 2000 rotate per minute (rpm) for 15 min. The supernatant part of each centrifuged sample was taken and filtered with a vacuum pump through GF/F Whatman^®^ filter papers (47 mm diameter and 0.7 µm pore size). Then, after adding 20 mL of sodium chloride (NaCl-Merck EMSURE^®^, Merck, Darmstadt, Germany) to the samples remaining in the centrifuge tube, they were centrifuged again at 2000 rpm for 15 min. Again, the supernatant of the centrifuged samples was taken and filtered through the same filter paper. A total of 20 mL of zinc chloride (ZnCl_2_-Merck EMSURE^®^, Germany) solution was added to the samples remaining in the centrifuge tubes. Then, these samples were centrifuged at 2000 rpm for 15 min and filtered through the same filter paper. This process was applied for all the fruit and vegetable samples. Each of the filter papers was placed in the Petri dishes separately, closed and left to dry at room temperature (23 °C).

Filter papers were examined under a stereo microscope (Leica^®^, Wetzlar, Germany) and the microplastics were counted and grouped according to their color, shape, and size. Microplastics were classified as red, blue, green, yellow, white, grey, black, and other in terms of color and as fragments, fibrils, film, and foam particles in terms of shape [66,67,68,69]. All the results were expressed as particles per gram (particle g^−1^).

### 2.4. SEM Analysis

HITACHI™ SU5000 field emission scanning electron microscope (FE-SEM) (HITACHI™, Tokyo, Japan), was used for the surfaces analysis of the microplastics for a subsample of the filters. The samples were dried for 24 h and then transferred to a stub and coated with gold sputtering to make it conductive. The surface of the sample was scanned with an Energy Dispersive Spectroscopy (EDS) detector (Oxford X-MaxN 80 mm^2^ detector, Oxford Instruments, Abingdon, UK) at 15 kV [70,71].

### 2.5. Polymers Characterization by ATR-FTIR

Polymer characterization of microplastics was carried out by using Attenuated Total Reflection Fourier Transform Infrared Spectroscopy (ATR-FTIR Thermo Scientific™ Nicolet iS10, Thermo Fisher Scientific, Waltham, MA, USA). Plastic particles were randomly selected from a subsample of the analyzed filters and carefully placed into a Petri plate using steel forceps and then processed by ATR-FTIR.

### 2.6. Risk Assessment

Estimated Daily Intake (EDI) values for each fruit and vegetable were calculated with the formula below [72,73].

EDI = (C × IR)/BW,(1)

where:

C: Mean number of microplastics per gram detected in sample tissue (items kg^−1^ day^−1^)

IR: The Daily Ingestion Rate per capita for pear (0.01 kg day^−1^), apple (0.09 kg day^−1^), tomato (0.30 kg day^−1^), onion (0.06 kg day^−1^), potatoes (0.14 kg day^−1^), and cucumber (0.05 kg day^−1^) in Turkey [74].

BW: Body weight 70 kg for adults and 16 kg for children [75].

Estimated Annual Intake (EAI) values of microplastics based on the consumption of fruit and vegetables were calculated using the following equation [49,76,77,78].

EAI = C × AIR,(2)

where:

C: Average number of microplastics detected per gram in fruit and vegetable tissues (particle g^−1^)

AIR: Annual Ingestion Rate per capita for pear (5202.5 g year^−1^), apple (31,166.6 g year^−1^), tomato (110,700.0 g year^−1^), onion (21,100.0 g year^−1^), potatoes (51,300.0 g year^−1^), and cucumber (18,500.0 g year^−1^) [74].

AIR per capita was calculated by dividing the amount of food item consumed in Turkey by the population (Population of Turkey: 83,614,362 [74]).

### 2.7. Statistical Analysis

All data were first gathered in Excel. Thus, all statistical analyses were accomplished using StatSoft^®^ Statistica STAT 10.0 software. This software is an integrated data analysis, graphics, featuring analytic procedures for science applications. First, the basic descriptive statistics (mean, minimum, maximum, and standard deviation) was calculated for each group (in term of the vegetable item, purchase site, color, shapes and size). Appropriate sample size and power calculation were previously determined using the G*Power 3.1 software using a large effect size (f = 0.40; α = 0.05, power = 0.7). Then, a comparison in the amount of microplastics among samples, among different purchase sites, in terms of color, shapes and size was accomplished by applying one-way analysis of variance (ANOVA). Hence, when the differences among the groups resulted significant, the significance of differences between pairs of group means was tested by post-hoc Tukey test A level of *p* < 0.05 was considered significant for all the analyses.

## 3. Results

### 3.1. Classification of Microplastic in Terms of Abundance

A total of 210 microplastics (average 2.9 ± 1.6 particles g^−1^) were detected in all samples (*n* = 72). The mean microplastics occurrence in the different products according to the purchase sites is presented in Table 1.

**Figure 1:**
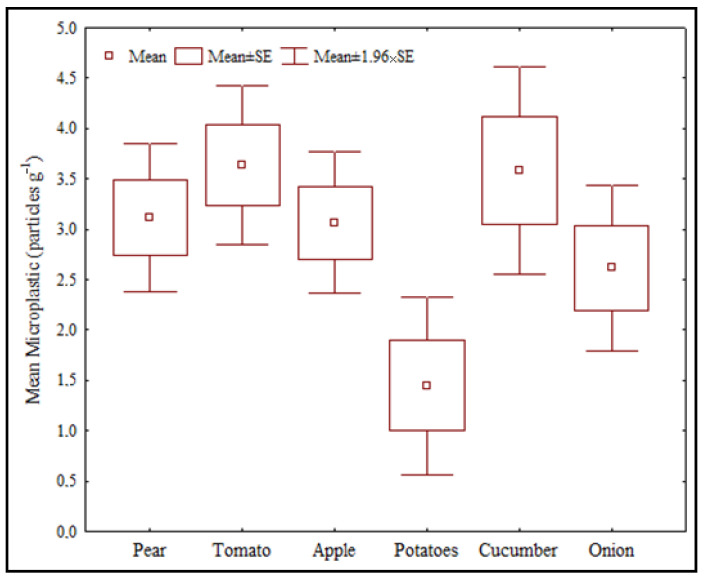
Microplastic occurrence in different fruits and vegetables.

The maximum amount of microplastics was determined as 44 in tomato samples, followed by cucumber (43 particles), pear (38 particles), apple (37 particles), onion (31 particles), and potatoes (17 particles). Statistically significant differences were determined between potato and tomato (*p* = 0.007) and between potato and cucumber (*p* = 0.009) (Figure 1).

Considering the different markets, the maximum amount of microplastic was detected in M1 (54 particles), followed by F1 (53 particles), F2 (52 particles), and M2 (51 particles), respectively. No statistical difference was determined between markets and fruiteries (*p* > 0.05).

**Figure 2:**
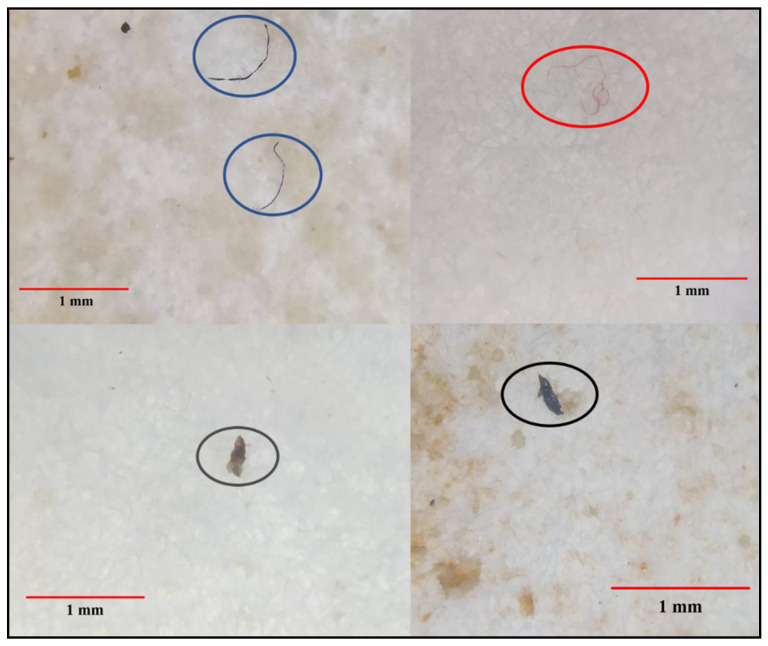
Some microplastics in different colours and shapes extracted from fruit and vegetable samples in this study.

The occurrence of microplastics in the fruits and vegetables samples in terms of color, shape, and size according to purchase sites is presented in Table S1 of the Supplementary Materials. A total of 59.3% of all microplastics were fragments followed by fibril (34.8%) and film (5.9%), respectively (Figure 2).

**Figure 3:**
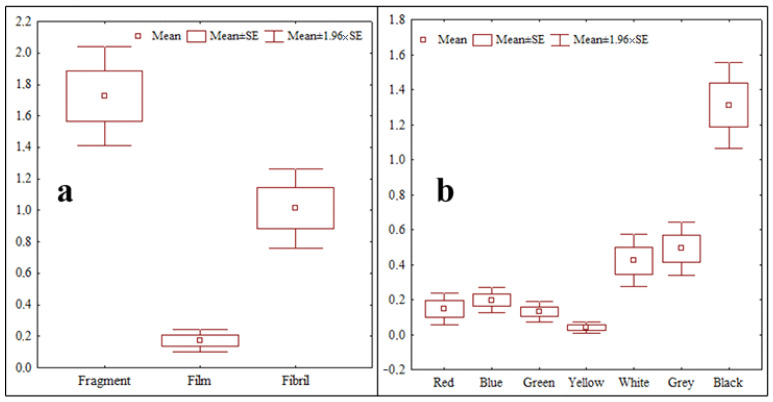
Statistical comparison of total MPs accumulation detected in fruits and vegetables in terms of shape and color ((**a**): shape, (**b**): color).

Statistically significant differences were determined between different shapes of microplastic (*p* < 0.05) (Figure 3a). Black colored microplastics were the dominant group (45.5% of samples), followed by grey (17.9%), white (16.5%), blue (7.8%), red (6.1%), green (4.5%), and yellow (1.7%), respectively. Statistically significant differences were determined between black-colored microplastics and all other color groups (*p* < 0.05) (Figure 3b). Microplastics in the 0.1 µm–1 mm size group were significantly more numerous (rate of 86.1%) than those of the 1–5 mm size group (*p* < 0.05).

**Figure 4:**
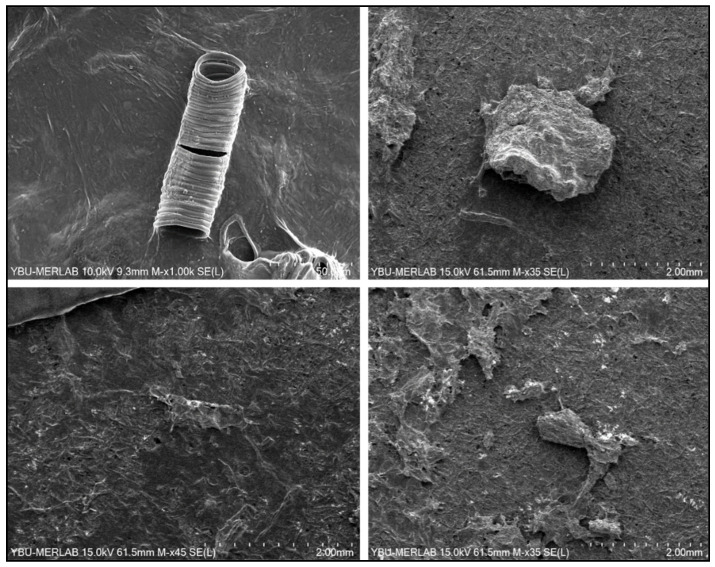
Microplastic images under a scanning electron microscope, at different magnifications.

Magnified images of the microplastics detected in the analysed samples by SEM-EDS are reported in Figure 4.

**Figure 5:**
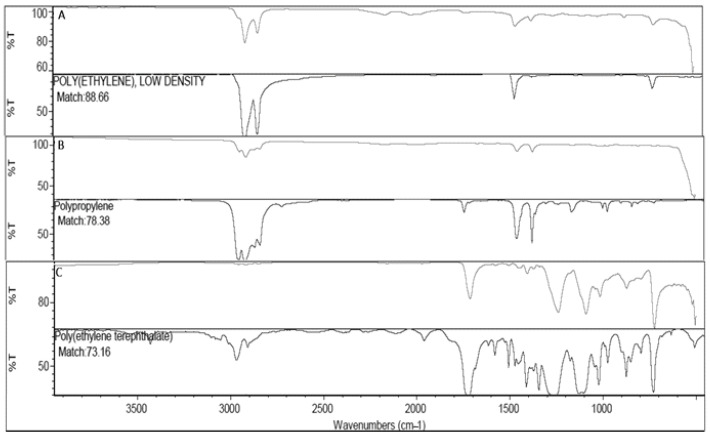
Results of the polymer’s characterization by ATR-FTIR. (**A**) Poly(ethylene), low density; (**B**) Polypropylene; (**C**) Poly(ethylene) terephthalate.

The polymers characterization by ATR-FTIR revealed the highest matchings with three main polymers: Polyethylene low density (PE) (in 60% of the samples; best match 88.66% and 79.47% avg.), Polypropylene (PP) (in 20% of the samples; best match 78.38% and 71.46% avg.) and Polyethylene terephthalate (PET) (in 20% of the samples; best match 73.16% and 70.02% avg.) (Figure 5).

### 3.2. Risk Assessment

According to risk analysis results, the highest EAI was determined in tomato samples as 398,520 particles individual^−1^ year^−1^. This was followed by apples, potatoes, cucumber, onion, and pears, respectively (Table 2). According to the results of EDI calculations, children ingest more microplastics through the consumption of fruits and vegetables than adults. The highest daily intake was found to be 68.24 particles kg^−1^ day^−1^ for children followed by adults (15.60 particles kg^−1^ day^−1)^ in tomato. With the consumption of 1 portion of 100 gr tomato, 6.8 particles (17.1 particles per 250 g portion) for children and 1.6 particles (3.9 particles per 250 g portion) for adults could enter the digestive system daily. Moreover, in the report published by [46], it is recommended to consume 400 g of fruits and vegetables daily. When tomatoes (the product with the highest amount of microplastics determined in the current study) are consumed in the amount recommended by [79], 2.76 particles for children, and 6.24 particles for adults can enter the digestive tract daily.

## 4. Discussion

It is well-recognized that microplastics are present in every environment constituting the biosphere and, accordingly, environmental pollution from micro- and nano-plastics has become an emerging problem. Plastics, which gain waste status after their usage is completed, may enter many urban and rural areas and, consequently, the soil where agricultural activities take place may be polluted. Microplastics can also reach agricultural areas (wastewater treatment plants, irrigation water, and atmospheric precipitation) directly or indirectly through the degradation of plastics used in agricultural activities [38,80,81]. This contamination in the soil naturally affects the fruits and vegetables grown in this environment. Many studies have reported that microplastics can move vertically deeper than the soil surface in various ways, such as farming activities, rhizome harvesting (e.g., potatoes, carrots), and cracks in the soil surface caused by dry climate [80,82,83,84]. Microplastics detected in fruits and vegetables may reach the plants through various factors during the cultivation of the crops. Microplastics reaching deep from the soil surface can be transported to various plant parts such as leaves, stems, and fruits after being accumulated in the roots [85,86,87]. Nevertheless, it is possible only for the particle of small size able to pass through the xylem.

The current study investigated the possible MP pollution in the most consumed fruits and vegetables (pear, apple, tomato, onion, potato, and cucumber) in Turkey and evaluated the potential risk deriving from their consumption in terms of public health. To this aim, samples of some of the most consumed fruits and vegetables in Turkey (pear, apple, tomato, onion, potato, and cucumber) were purchased from four different markets and fruiterer in Muğla province. A total of 210 microplastics (average 2.9 ± 1.6 particles g^−1^) were detected in all samples (*n* = 72). The average microplastic number detected in fruits per gram was apple 3.1 ± 1.2 and pear 3.1 ± 1.3. The average occurrence in vegetables per gram were tomato 3.6 ± 1.4, cucumber 3.6 ± 1.8, onion 2.6 ± 1.5, and potato 1.5 ± 1.6. Since there are a few studies on the presence of microplastics in agricultural areas and especially their accumulation in fruits and vegetables, the results obtained from the current study could be compared only with a limited number of studies [57], investigating the accumulation of nano and microplastics in the edible parts of different fruits and vegetables (apple, pear, broccoli, lettuce, and carrot) purchased in different markets in Catania (Italy), reported that the nano- and microplastic amounts was: 2.0 × 10^5^ ± 1.3 × 10^5^ in apple, 1.9 × 10^5^ ± 1.1 × 10^5^ in pear, 1.3 × 10^5^ ± 0.8 × 10^5^ in broccoli, 5.1 × 10^4^ ± 2.5 × 10^4^ in lettuce, and 1.0 × 10^5^ ± 0.4 × 10^5^ in carrot. The results reported by [57] substantially higher than the findings of this current study because, together with microplastics, they also investigated the possible accumulation of nanoplastics in the edible tissues of examined fruits and vegetables. While 86.1% of microplastics detected in our study are in the 0.1–1000 µm size group (the other group’s size is larger), the average size of the plastic particles detected in [57] ranged from 1.51 to 2.52 µm. Ref. [59] analyzed the presence of microplastics in fruits (grapes and banana) and vegetables (brinjal and potato) samples taken from different markets in Trichy, Tamil Nadu (India) and reported the occurrence of microplastics of 2 and 10 µm size in fruits and 2 and 10 µm size in vegetables, respectively. Ref. [58] detected 1 μm and 0.2 μm sized polystyrene (PS) microplastics in carrot roots and leaves, respectively. All these studies showed that humans can be directly exposed to microplastics through fresh fruits and vegetables.

Although our knowledge about the interference of microplastics from soil to different tissues of plants is still limited, transpiration pull has a significant role in plant uptake and bioaccumulation of plastic particles [85,88]. Experimental studies in fully controlled environments have proven that plants can carry nanoscale (<100 nm), submicrometer (<1 μm), and micro-sized (≥1 μm) plastics from their roots to their leaves [38,58,89,90,91]. Ref. [41] stated that plastic particles entered the epidermal tissue of wheat roots and are stimulated via the pericycle and transported into the xylem. They also reported that the particles could pass through the xylem to the aerial part of the plant. In addition, Ref. [91] reported that after the accumulation of microplastics in the root of cucumber, they could be transported to leaves, flowers, and fruits through the stems. However, in almost all of these studies on translocation of microplastics, nanoscale plastics (<100 nm) were primarily examined, while micro-sized plastics (1 or 2 μm) remained in the roots. In the current study, plastics were counted under the microscope and classified according to their size and color. Although 86.1% of the plastics detected were in the range of 0.1–1000 µm, these measurements were made on only visible particles. This is one of the limitations of the current study. Previous studies showed that in such measurements made with the naked eye using a microscope, particles below certain sizes could not be distinguished from each other and even could not be seen. For instance, Ref. [92] stated that sizes below 500 µm cannot be distinguished and classified in the counts made with the naked eye under the microscope. Also, Ref. [93] emphasized that it was problematic for the human eye to identify microplastics with a size of 200 µm under the microscope. In this sense, although the plastics detected in this study were determined as 0.1–1000 µm by definition, they were particles closer to 1 mm in size and it is impossible that particles of this size could reach the plant tissues from the soil by direct absorption. Previous studies showed that micro- and nano-plastics could adhere to the leaves of plants. In a previous study, a solution containing 100 and 500 nm (average particle size of micronanoplastics’ (MNP) 105.53 ± 3 nm and 532.06 ± 26 nm, respectively) polystyrene microplastics were sprayed onto lettuce leaves in the growing stage. Leaves were then subjected to multiple washings and after microplastics treatment, a large amount of these particles accumulated on the lettuce leaves were still detected on the tissues [94].

Considering the polymer characterization, the most dominant group determined as a result of the FTIR analysis in the current study was PE (60%) followed by PP and PET (20% and 20%, respectively). Plastic-based materials are commonly used in packaging, transportation, storage, and exhibit, especially in markets, which are among the suppliers for food products to reach the end consumer, and PE and PP are the most commonly used plastic types [95]. Plastic packaging is an important method for keeping fruits and vegetables fresh and store them to the consumers fresh as they have been harvested from the field. For example, the most effective storage conditions for the fruits and vegetables used in this study are polyethylene bags, plastic, polystyrene, cardboard trays, polyethylene, or polypropylene flow wrapping for apples, polyethylene bags for potatoes, plastic punnets covered in film or flow wrapping for tomatoes [96], PP non-perforated packaging for pears, PE bags and net bags for onions [97], and shrink-wrapped plastic packaging for cucumbers [98]. Thus, petroleum-derived plastic materials are used extensively in all of these storage conditions. Contamination of food items from plastic packaging has been already reported by [99] investigating food delivery and disposable plastic cups for daily drinking. Thus, it is highly possible that the plastic particles eroded from the packaging materials contaminate the stored fruits and vegetables: the soft surface of the examined vegetable (such as tomatoes or cucumbers) can be easily damaged by physical impact with the packaging during transport. Considering the size of the particles detected in the edible parts of fruits and vegetables in the current study, it is highly likely that microplastics reached the sample tissues as a result of contamination during the storage processes instead of relocation from the plant’s transport system.

With regard to the risk assessments, it was determined that children are more exposed to microplastics due to their higher rate of consumption of fruits and vegetables. The tolerable daily intake (TDI) for plastics has not yet been determined. Therefore, it is not possible to determine whether this level of exposure complies with regulations. However, in the report published by [100], in order to see the worst-case scenario in terms of public health risk assessment, it is stated that one portion (250 g) can contain up to 1000 microplastic particles considering the highest reported concentration of microplastics (4 particles g^−1^) in mussels. All the EAI and EDI values determined in the current study are much lower than this upper limit. However, it may pose a risk to human health as there is no tolerable limit value for plastics. Ref. [57] reported that the maximum EDI values for children and adults for apple samples (1.41 × 10^6^ particles kg day^−1^ for children, 4.62 × 10^5^ particles kg day^−1^ for adults) whereas EDI values for pear samples were 1.37 × 10^6^ particles kg day^−1^ for children and 4.48 × 10^5^ particles kg day^−1^ for adults. As mentioned above, the discrepancy between the results of that study and the current can be due to the calculation of both nano- and microplastic numbers in their study. Ref. [101] reported the EDI values for microplastics in mineral waters in plastic bottles as 1.5 × 10^6^ p^−1^ kg^−1^ body-weight^−1^ day^−1^ and 3.4 × 10^6^ p^−1^ kg^−1^ body-weight^−1^ day^−1^ for adults and children, respectively. These results are much higher than the EDI values found in the current study (Table 2).

## 5. Conclusions

This study reveals the presence of microplastics in the edible parts of the mostly consumed fresh vegetables and fruits in the Turkish market. Although the analyzed samples were collected only from a single region, the study represents the first reference for Turkey and one of the few available studies focusing on the occurrence of microplastics in vegetables and fruits. The occurrence of microplastics of big size, that are not allowed to pass by xylem transport, suggests that fresh vegetables and fruits can be contaminated with plastic, especially during the production phase, during agricultural activities (greenhouses, plastic crates, additive fertilizers, etc.) and during the marketing process (transport to the market and purchasing process). Nevertheless, in nutritional diets recommended for healthy eating all over the world (especially the World Health Organization’s (WHO) Mediterranean diet) at least 400 g of fruits and vegetables must be consumed every day to maintain good health [102,103]. Nonetheless, it would be a mistake to ignore this serious food safety issue due to the lack of information on how much microplastics are emitted, whether by conventional, integrated or organic means. Therefore, it is extremely important to monitor the quality of fruits and vegetables and identify and minimize the potential sources of contamination that can occur during the food supply chain. Considering the broad presence of microplastics and the lack of certain regulations, the development of standard methodologies for fruits and vegetables is highly suggested.

## Figures and Tables

**Table 1:** Mean occurrence of microplastics in fruits and vegetables for each purchase site (mean ± standard deviation particles g^−1^).

Purchase Site	Product						
	Pear	Tomato	Apple	Potatoes	Cucumber	Onion	Mean
M_1_	3.7 ± 0.6	3.3 ± 2.1	3.5 ± 0.7	0.5 ± 0.5	3.5 ± 3.3	3.5 ± 2.3	3.0 ± 2
M_2_	3.0 ± 0.7	3.7 ± 0.5	2.2 ± 0.7	1.0 ± 0.8	4.1 ± 0.8	2.8 ± 0.6	2.8 ± 1.2
F_1_	3.6 ± 0.5	4.9 ± 0.8	3.5 ± 2.2	1.0 ± 0.7	2.1 ± 1.1	2.5 ± 1.7	2.9 ± 1.7
F_2_	2.2 ± 2.4	2.5 ± 0.8	3 ± 0.9	3.3 ± 2.1	4.6 ± 0.9	1.7 ± 0.8	2.9 ± 1.6
Mean	3.1 ± 1.3	3.6 ± 1.4	3.1 ± 1.2	1.5 ± 1.6	3.6 ± 1.8	2.6 ± 1.5	2.9 ± 1.6

**Table 2:** Estimated annual and daily intake amounts (EAI and EDI) of the microplastics related to the consumption of fruits and vegetables.

	EAI (Particles Individual^−1^ Year^−1^)	EDI (Particles kg^−1^ Day^−1^)	
		Children	Adult
Pear	16,127.6	2.76	0.63
Apple	96,617.4	16.54	3.78
Tomato	398,520.0	68.24	15.60
Onion	54,860.0	9.39	2.15
Potatoes	76,950.0	13.18	3.01
Cucumber	66,600.0	11.40	2.61

## Data Availability

The data used during the current study are available from the corresponding author on a reasonable request.

## Supplementary Materials

The following supporting information can be downloaded at: https://www.mdpi.com/article/10.3390/life13081686/s1. Table S1: The abundance of microplastics in the examined samples of fruits and vegetables in terms of shape, color, and size by purchase sites (mean ± SD particles g^−1^).
