# Supplementary material for: Occurrence of Microplastics in Most Consumed Fruits and Vegetables from Turkey and Public Risk Assessment for Consumers
Source: Life (Basel). 2023 Aug 4;13(8):1686. doi: 10.3390/life13081686 (PMC10455475; doi:10.3390/life13081686)
Supplement: Supplementary file 1 [file life-13-01686-s001.zip › life-2493557-supplementary.pdf]

Table S1: Abundance of microplastics in the examined samples of fruits and vegatables in terms of shape, color and size by purchase sites (mean ± SD particles g^{-1})

		Product																							
		Pear				Tomato				Apple				Potatoes				Cucumber				Onion			
		M1	M2	F1	F2	M1	M2	F1	F2	M1	M2	F1	F2	M1	M2	F1	F2	M1	M2	F1	F2	M1	M2	F1	F2
**Shape**	Fragment	2.4 ± 1.7	2.4 ± 0.5	2.7 ± 1.0	1.3 ± 2.1	0.7 ± 0.3	2.9 ± 0.9	4.3 ± 0.8	0.8 ± 0.8	1.3 ± 1.6	1.6 ± 1.1	2.6 ± 1.5	2.4 ± 0.9	0.1 ± 0.2	0.4 ± 0.4	0.8 ± 0.5	1.7 ± 1.5	0.3 ± 0.2	2.2 ± 0.3	1.3 ± 1.0	3.6 ± 0.9	1.0 ± 0.0	2.3 ± 0.7	1.7 ± 1.1	0.5 ± 0.4
	Film	0.1 ± 0.1	0.1 ± 0.1	-	0.3 ± 0.3	0.2 ± 0.3	0.1 ± 0.1	0.2 ± 0.3	0.7 ± 0.4	0.2 ± 0.2	-	0.6 ± 1.0	-	-	0.1 ± 0.1	0.1 ± 0.1	0.4 ± 0.5	0.2 ± 0.2	-	0.1 ± 0.1	0.1 ± 0.1	0.3 ± 0.3	0.1 ± 0.2	0.1 ± 0.1	0.2 ± 0.2
	Fibril	1.1 ± 1.5	0.5 ± 0.2	0.9 ± 0.6	0.6 ± 0.0	2.5 ± 1.6	0.7 ± 0.4	0.4 ± 0.2	1.0 ± 0.2	2.0 ± 0.9	0.6 ± 0.4	0.3 ± 0.3	0.6 ± 0.0	0.3 ± 0.3	0.5 ± 0.6	0.1 ± 0.1	1.2 ± 0.9	3.1 ± 2.8	1.9 ± 0.6	0.7 ± 0.3	0.9 ± 0.8	2.2 ± 2.2	0.4 ± 0.2	0.7 ± 0.5	1.0 ± 0.3
**Colour**	Red	0.1 ± 0.1	-	-	-	-	0.1 ± 0.1	-	0.4 ± 0.7	-	0.1 ± 0.1	-	-	-	0.1 ± 0.1	-	0.3 ± 0.2	0.1 ± 0.1	0.2 ± 0.2	-	1.1 ± 1.3	0.7 ± 0.8	-	0.1 ± 0.1	0.2 ± 0.2
	Blue	0.3 ± 0.6	0.1 ± 0.1	0.3 ± 0.3	-	0.8 ± 0.4	0.3 ± 0.1	0.1 ± 0.1	0.3 ± 0.2	0.3 ± 0.1	-	-	-	0.1 ± 0.1	-	0.1 ± 0.1	0.4 ± 0.5	0.4 ± 0.4	0.3 ± 0.3	-	0.2 ± 0.2	0.7 ± 0.6	-	-	0.1 ± 0.1
	Green	-	0.1 ± 0.1	0.1 ± 0.1	0.1 ± 0.1	0.3 ± 0.3	0.1 ± 0.1	0.1 ± 0.2	-	-	-	0.2 ± 0.3	-	0.1 ± 0.1	-	-	0.1 ± 0.2	0.7 ± 0.7	0.1 ± 0.2	0.1 ± 0.2	0.1 ± 0.1	0.3 ± 0.2	0.1 ± 0.1	0.4 ± 0.4	0.2 ± 0.2
	Yellow	-	0.1 ± 0.1	-	0.1 ± 0.1	0.1 ± 0.1	0.1 ± 0.1	-	-	0.1 ± 0.2	-	-	-	0.1 ± 0.1	0.1 ± 0.1	-	-	0.3 ± 0.6	-	-	0.1 ± 0.1	-	-	-	-
	White	0.5 ± 0.5	0.2 ± 0.2	0.2 ± 0.3	0.7 ± 1.3	0.3 ± 0.6	0.1 ± 0.1	1.7 ± 0.6	-	0.4 ± 0.2	0.4 ± 0.2	0.7 ± 1.0	0.3 ± 0.6	0.1 ± 0.2	0.3 ± 0.3	0.1 ± 0.1	1.5 ± 1.6	0.7 ± 0.6	0.1 ± 0.1	0.1 ± 0.1	1.0 ± 0.7	-	0.1 ± 0.2	0.3 ± 0.3	0.3 ± 0.1
	Grey	0.3 ± 0.3	0.1 ± 0.1	0.3 ± 0.3	0.9 ± 1.2	1.1 ± 0.8	0.1 ± 0.2	1.4 ± 1.1	0.7 ± 0.8	0.7 ± 0.9	0.2 ± 0.2	0.9 ± 0.7	1.8 ± 0.9	0.1 ± 0.1	0.3 ± 0.3	-	0.2 ± 0.2	0.4 ± 0.7	0.6 ± 0.3	0.3 ± 0.2	0.3 ± 0.2	0.8 ± 0.8	0.2 ± 0.3	0.1 ± 0.1	0.1 ± 0.1
	Black	2.4 ± 1.2	2.2 ± 0.5	2.6 ± 1.1	0.5 ± 0.1	0.7 ± 1.0	2.9 ± 0.8	1.4 ± 1.2	0.7 ± 0.3	1.9 ± 0.2	1.5 ± 1.0	0.9 ± 1.5	0.9 ± 0.6	-	0.3 ± 0.3	0.8 ± 0.5	0.7 ± 0.5	0.5 ± 0.8	2.8 ± 0.6	1.5 ± 0.7	0.9 ± 0.8	0.9 ± 0.5	2.3 ± 0.4	1.5 ± 1.3	0.9 ± 0.6
**Size**	0.1 μ - 1 mm	3.2 ± 0.8	2.9 ± 0.6	3.5 ± 0.6	2 ± 2.4	2.2 ± 1.5	3.3 ± 0.8	4.7 ± 0.9	1.7 ± 1.3	2.6 ± 0.7	1.9 ± 0.8	3.4 ± 2.2	2.7 ± 1.0	0.3 ± 0.4	1.0 ± 0.8	0.9 ± 0.7	2.5 ± 1.7	2.5 ± 3.3	3.7 ± 0.8	2.0 ± 1.1	4.3 ± 0.8	2.3 ± 1.2	2.6 ± 0.5	2.2 ± 1.4	1.5 ± 0.9
	1 mm - 5 mm	0.5 ± 0.5	0.1 ± 0.1	0.1 ± 0.1	0.2 ± 0.0	1.1 ± 0.6	0.5 ± 0.3	0.2 ± 0.2	0.8 ± 0.6	0.9 ± 0.2	0.3 ± 0.1	0.1 ± 0.2	0.3 ± 0.1	0.1 ± 0.1	-	0.1 ± 0.1	0.8 ± 0.4	1.0 ± 1.6	0.3 ± 0.1	0.1 ± 0.1	0.3 ± 0.1	1.1 ± 1.8	0.2 ± 0.2	0.3 ± 0.5	0.3 ± 0.1
**Total**[*]		3.7 ± 0.6	3.0 ± 0.7	3.6 ± 0.5	2.2 ± 2.4	3.3 ± 2.1	3.7 ± 0.5	4.9 ± 0.8	2.5 ± 0.8	3.5 ± 0.7	2.2 ± 0.7	3.5 ± 2.2	3.0 ± 0.9	0.5 ± 0.5	1.0 ± 0.8	1.0 ± 0.7	3.3 ± 2.1	3.5 ± 3.3	4.1 ± 0.8	2.1 ± 1.1	4.6 ± 0.9	3.5 ± 2.3	2.8 ± 0.6	2.5 ± 1.7	1.7 ± 0.8

[*]Total values represent the same values for shape, color and size groups
